# Transvaginal natural orifice transluminal endoscopic surgery for early-stage ovarian cancer and borderline ovarian tumors: a case series

**DOI:** 10.3389/fsurg.2025.1542486

**Published:** 2025-02-19

**Authors:** Gaétan Kellerhals, James Nef, Yannick Hurni, Daniela Huber

**Affiliations:** ^1^ Faculty of Medicine, University of Geneva, Geneva, Switzerland; ^2^ Department of Gynecology and Obstetrics, Sion Hospital, Sion, Switzerland; ^3^ Department of Pediatrics, Gynecology and Obstetrics, Geneva University Hospitals, Geneva, Switzerland

**Keywords:** early-stage ovarian cancer, non-epithelial ovarian cancer, borderline ovarian cancer, natural orifice transluminal endoscopic surgery, vNOTES, minimally invasive surgery, fertility-sparing surgery, ovarian cancer treatment

## Abstract

**Introduction:**

Surgery is the cornerstone of ovarian cancer treatment. Transvaginal natural orifice transluminal endoscopic surgery (vNOTES) is a novel, minimally invasive technique that is gaining interest in gynecological oncology. However, its use in ovarian cancer is still limited, with only a few cases reported. This study aimed to evaluate the feasibility of vNOTES for the surgical staging of borderline and early-stage ovarian cancer.

**Methods:**

We retrospectively reviewed all cases of borderline ovarian tumors (BOTs) and early-stage ovarian cancer surgically staged by vNOTES at our institution between October 2021 and August 2024.

**Results:**

Eleven patients were included, seven with early-stage ovarian or tubal cancer and 4 with BOTs. The median age was 47 (27–81) years, and the median body mass index was 28.1 (22.4–39.2) kg/m^2^. Complete vNOTES staging was achieved in all cases, including peritoneal washing, unilateral/bilateral salpingo-oophorectomy, abdominal cavity inspection, peritoneal biopsies, infracolic omentectomy, and total hysterectomy when required. The median operating time was 70 (35–138) min, with a median blood loss of 50 (10–100) ml. No intraoperative complications occurred except for one case of minor ovarian spillage. No conversions to conventional laparoscopy or laparotomy were needed. Postoperative complications included one surgical site infection (9.1%) and 2 cases of postoperative cystitis (18.2%). No severe complications graded ≥3 on the Clavien-Dindo classification were observed.

**Conclusion:**

vNOTES appears to be a feasible approach for the surgical staging of highly selected patients with early-stage adnexal malignancies. Further studies are needed to validate its long-term safety and oncological outcomes.

## 1 Introduction

Surgery remains the cornerstone of ovarian cancer treatment, with the primary goal being the complete resection of the tumor. The quality of surgery and the surgeon's expertise are critical to patient outcomes and survival. With advances in surgical techniques and a growing focus on improving patient perioperative outcomes, minimally invasive surgery (MIS) has become increasingly important in managing gynecological malignancies. However, its application in the treatment of ovarian cancer remains a subject of on-going debate.

Current guidelines recommend performing cytoreductive surgery for ovarian cancer via midline laparotomy, even in its early stages (1). However, to date, no randomized controlled trials have directly compared MIS with open surgery for the treatment of early-stage ovarian cancer and borderline ovarian tumors (BOTs) (2). Several studies suggest the feasibility and safety of MIS approaches for the management of early-stage ovarian cancer, appearing to be non-inferior to laparotomy (3, 4) and presenting with lower rates of surgical complications (5, 6).

Transvaginal Natural Orifice Transluminal Endoscopic Surgery (vNOTES) is an innovative, minimally invasive approach that combines laparoscopy and vaginal surgery (7). This approach has proven its feasibility and safety for treating several benign gynecological conditions, being a valuable option for performing hysterectomies, myomectomies, adnexal procedures, and pelvic organ prolapse treatments with a short learning curve (7–12). In addition, vNOTES has shown promising results in managing early-stage endometrial cancer, allowing complete surgical staging, including sentinel lymph node biopsies, lymphadenectomies, and omentectomies (10, 13–16). However, although increasing evidence supports the use of vNOTES approaches to manage early-stage endometrial cancer and to perform benign adnexal surgeries, little is known about the feasibility and safety of performing vNOTES oncological staging for tubo-ovarian malignancies (14, 17–19). Hereby, we report our initial experience performing vNOTES surgical staging for early-stage ovarian cancer and BOTs.

## 2 Materials and methods

### 2.1 Patient selection, data collection, and methods

vNOTES was implemented in our institution in May 2020. Since January 2022, we have collected retrospectively and prospectively data concerning patients who underwent vNOTES procedures to create an institutional database using the Research Electronic Data Capture (REDCap) software. The project received approval from the local ethical committee (Commission cantonale d'éthique de la recherche sur l'être humain, CER-VD), with registration number 2021-02346, and all patients gave written informed consent.

From this database, we retrospectively identified all patients diagnosed with borderline ovarian tumors (BOTs) or early-stage ovarian cancer between May 2020 and August 2024. At our institution, exclusion criteria for a vNOTES approach include confirmed ovarian cancer, active genital tract infections, history of rectovaginal endometriosis, rectal surgery, pelvic radiotherapy, severe pelvic inflammatory disease and mesh sacrocolpopexy. All patients underwent pelvic ultrasound, thoraco-abdominal computed tomography, and tumor marker levels. In addition, pelvic magnetic resonance imaging (MRI) was performed when further characterization of ovarian lesions was necessary according to the recommendations of the European Society of Gynecologic Oncology (ESGO) (20). No patients showed evidence of advanced ovarian disease at the preoperative workup.

Demographic features, as well as clinical and perioperative information, were collected and analyzed. Intraoperative parameters included total operative time (from catheterization of the bladder to vaginal closure), vNOTES port insertion time (from incision to intrabdominal CO2 insufflation), estimated blood loss, intraoperative complications (including transfusion-requiring bleeding or iatrogenic organ injury), and the necessity for conversion to conventional laparoscopy or laparotomy. Postoperative assessments comprised pain evaluation using the visual analog scale graded from 0 to 10 at 12-, 24-, and 48-h post-surgery, opioid analgesic use, duration of hospital stay, and postoperative complications within 8 postoperative weeks, graded according to the Clavien-Dindo classification (CD) (21). In addition, we recorded histopathological results, the timing and type of any adjuvant therapies, and the patient status at the last follow-up.

Continuous variables were expressed as median and range, while dichotomous variables were represented as absolute numbers and percentages (%). No statistical inter-group comparisons were undertaken. Statistical analyses were performed using IBM SPSS version 29.0.2.0.

### 2.2 Surgical technique

All interventions were performed by the same oncogynecological surgeon (DH). Patients received a single dose of clindamycin vaginal cream 2% (5 g of cream with 100 mg of clindamycin) the day before the surgery, and 2–4 h before the intervention, in addition to cefuroxime 1.5 g (3 g for patients weighing more than 80 kg) and metronidazole 500 mg intravenously at induction of anesthesia. Under general anesthesia and muscular relaxation, patients were positioned in a horizontal dorsal lithotomy position, and a bladder catheter was placed.

**Figure 1:**
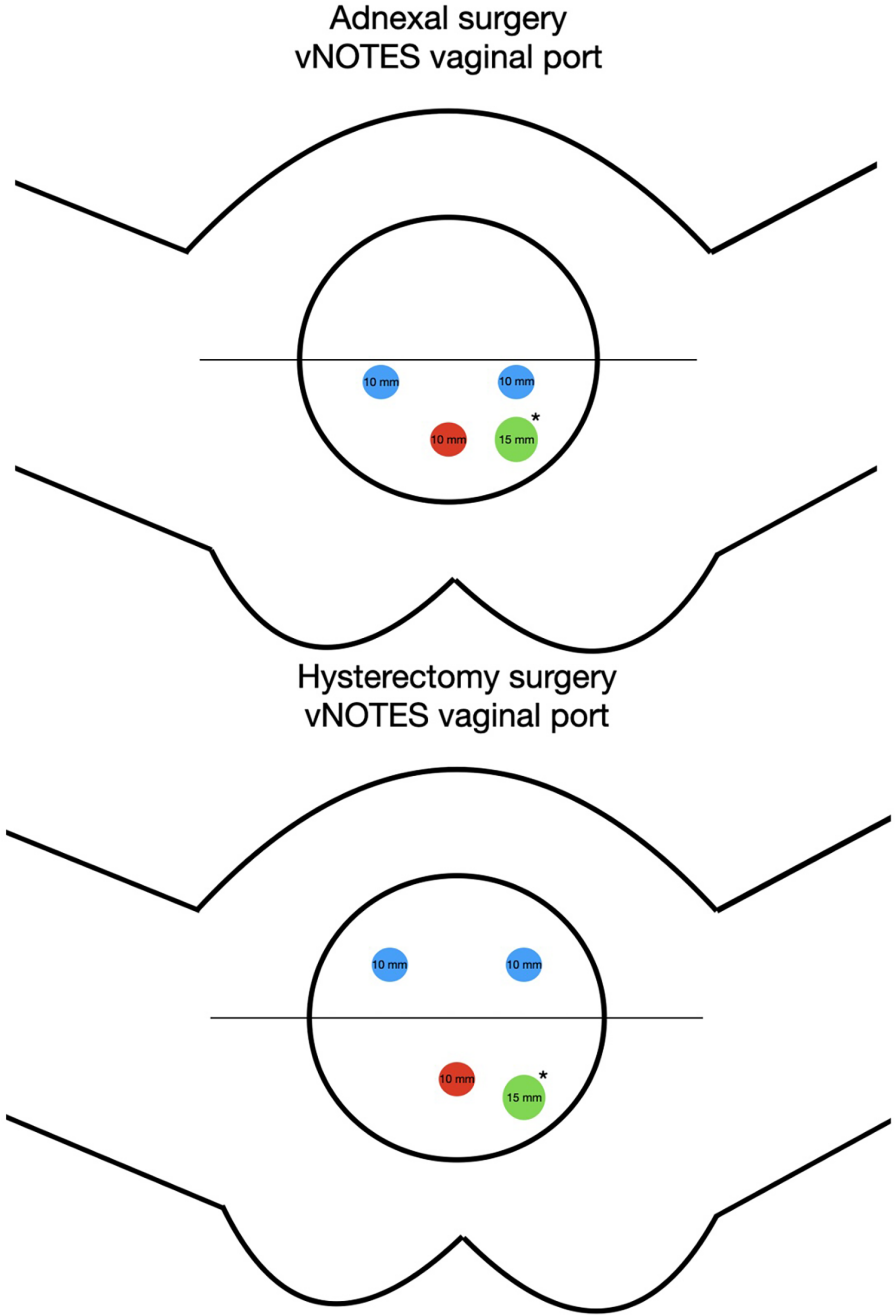
Adnexal surgery: blue = 10 mm trocar for instruments, Red = 10 mm optical trocar. The instrument trocars are inserted below the medial line for better access to the adnexa. Hysterectomy surgery: Blue = 10 mm trocar for instruments, Red = 10 mm optical trocar. The instrument trocars are inserted over the medial line. Green* = A 12 mm accessory trocar can be added as needed for exposure or insertion of an endobag.

Access was gained with a posterior 2 cm colpotomy through Douglas's pouch to perform interventions limited to the adnexa. If hysterectomies were performed, access to the abdominal cavity was achieved through anterior and posterior colpotomies, with the transvaginal uterosacral ligaments section when developing the posterior access. A vNOTES port (GelPoint vPath, Applied Medical, Rancho Santa Margarita, CA, USA) with an adapted diameter (7 cm for adnexectomies and 9.5 cm for hysterectomies) was placed in the abdominal cavity through the anterior and/or posterior colpotomies. Carbon dioxide was insufflated to create a pneumoperitoneum with an intraperitoneal pressure of 8–15 mmHg. Three 10 mm trocars were used to insert a 10-mm rigid 30° scope, 5-mm instruments such as Johan and bipolar graspers, and sealing devices. If necessary, a 4th 12 mm supplementary trocar was added (Figure 1).

Surgical staging included peritoneal washing, uni or bilateral salpingo-oophorectomy, abdominal cavity inspection, peritoneal biopsies, infracolic omentectomy, and total hysterectomy. In selected cases, fertility-sparing approaches with unilateral salpingo-oophorectomy or cystectomy and uterus preservation were performed. To perform hysterectomies, the uterine vessels, broad ligaments, and round ligaments were sealed and cut from caudal to cranial. Salpingo-oophorectomies were always performed after correctly visualizing the ureters, the fallopian tubes, and the infundibulopelvic ligaments, with utmost care to avoid spillage.

All specimens have been extracted vaginally. To avoid intraabdominal spillage, large adnexal lesions were retrieved into an Inzii Retrieval System of 10 or 15 cm of diameter, or Alexis Contained Extraction System of 14 or 17 cm (Applied Medical, Rancho Santa Margarita, CA, USA). Intraoperative frozen section analysis was performed in cases with suspicious adnexal masses. Omentectomies were performed with an articulating sealing device, as we previously described (14).

At the end of the procedure, the colpotomy was closed under direct visualization with a continuous Vicryl 0 suture, incorporating the anterior and posterior peritoneal folds and vaginal layers into the single running suture. Postoperatively, patients received a single dose of clindamycin vaginal cream 2% (5 g of cream with 100 mg of clindamycin) once a day during the first seven postoperative days.

## 3 Results

From October 2021 to August 2024, 7 patients with early-stage tubal or ovarian cancer and 4 patients with BOTs underwent surgical staging by vNOTES at Valais Hospital (Sion, Switzerland).

The median age was 47 (27–81) years, with a median body mass index of 28.1 (22.4–39.2) kg/m^2^. Seven patients (63.6%) were classified as American Society of Anesthesiologists score (ASA) II and four (36.4%) as ASA III. Table 1 provides an overview of patient characteristics and their perioperative outcomes.

**Table 1:** Patient characteristics and perioperative outcomes.

No.	Age	Comorbidities	Prior abdominal surgery	Surgery indication	ASA score	Ovarian size (mm)	Frozen section	Definitive histology	TNM	FIGO	Status last FU	FU (month)
1	81	Non Hodking lymphoma, Diabetes II, Hypothyroidism, Diffuse large B-cell lymphoma (DLBCL, NOS) of the terminal ileum	Right colectomy and ileal resection, ileotransverse anastomosis for diffuse large B-cell lymphoma	1 year persistent bilateral adnexal mass	3	41	BOT	Low grade serous carcinoma right ovary	pT2bpNx	IIB	NED	39
2	45	Depression, Obesity	Left adnexectomy	Prophylactic right adnexal surgery	2	-	No	15 mm adult granulosa cell tumor right ovary Implant rectal mesentery	pT2bpNx	IIB	NED	37.6
3	76	Pancreatic cancer, Diabetes Hypothyroidism	Whipple procedure for pancreatic cancer	Ovarian metastasis pancreatic cancer vs BOT	3	120	BOT	Mucinous BOT, peritoneal implants and washing positive for pancreatic cancer	pT1a	IA	DOC	11.1
4	44	Obesity	Caesarean section	Parasitic fibroma vs fibro sarcoma	2	60	No	High grade serous carcinoma right tube	pT2aNx	IIA	NED	29.2
5	60	–	TVT urinary mesh	Hysterectomy for metrorragia with benign endometrial hyperplasia	2	–	No	12 mm adult granulosa cell tumor right ovary	pT1a	IA	NED	34
6	27	Obesity	–	Bilateral ovarian teratoma	2	45	No	3 mm intracystic immature teratoma right ovary	pT1a	IA	NED	16.5
7	33	–	Laparoscopic ovarian cystectomy	Persistent cyst of benign appearance in the left ovary	2	40	BOT^a^	1 mm residual serous BOT left ovary	pT1c1	IC1	NED	13.4
8	47	Hypothyroidism	–	Suspected benign mucinous tumor	2	100	BOT	Invasive mucinous ovarian carcinoma with 2 mm infiltrating component	pT1c1	IC1	NED	12.5
9	76	Coronaropathy Obesity Endometrioid carcinoma	Appendectomy Hysterectomy, BSO and SLNB	Left tubal STIC	3	–	STIC^a^	Left tubal STIC	pT1a	IA	NED	10
10	50	–	–	7 cm right ovarian mass	2	70	BOT	Right serous BOT	pT1c3	IC1	NED	8.1
11	45	Bipolar disorder Diabetes II Hypertension Hypercholesterolemia	Caesarean section	6 months persistent left ovarian cyst	3	45	BOT	Serous BOT	pT1a	IA	NED	6

BOT, borderline ovarian tumors; DOC, died of other cause; TVT, tension-free vaginal urinary mesh; STIC, serous tubal intraepithelial carcinoma; BSO, bilateral salpingo-oophorectomy; SLNB, sentinel lymph node biopsy.

^a^ Histology from previous surgery.

Bilateral salpingo-oophorectomy was performed in five patients (45.5%), while six patients (54.5%) underwent fertility-sparing surgery with preservation of at least one ovary and the uterus. Table 2 summarizes the surgical procedures performed to complete surgical staging. The median operating time was 70 (35–138) min, with a median blood loss of 50 (10–100) ml. No conversion to conventional laparoscopy or laparotomy was necessary, and all procedures were performed as planned. In a patient with one suspicious pelvic implant, a hybrid approach was used to explore the utero-vesical peritoneum. All surgical material was extracted vaginally with an endobag. No intraoperative complications were reported, except for one case involving a minimal pelvic ovarian spillage during extraction in the retrieval system (9.1%). The percentage of patients with positive peritoneal cytology was 54.5% (Table 2).

**Table 2:** Surgical procedure, operative characteristics, and perioperative outcomes.

	Total, number (%)
Procedures performed	
Unilateral/bilateral salpingo-oophorectomy	11 (100)
Peritoneal washing	11 (100)
Infracolic omentectomy	9 (81.8)
Pelvic peritonectomy	3 (27.3)
Rectal mesenteric implant excision	1 (9.1)
Total hysterectomy	5 (45.5)
Adnexal diameter (mm)	45 (12–120)
Operative time (min)	70 (35–138)
Estimated blood loss (ml)	50 (10–100)
Hybrid access	1 (9.1)
Positive Peritoneal cytology	6 (54.5)
Perioperative complications	
Tumor spillage	1 (9.1)
Surgical site infection	1 (9.1)
Cystitis	2 (18.2)
Gastric ulcer and biliary pancreatitis	1 (9.1)
Length of stay (days)	2 (1–4)

Data are presented as median (range) or absolute number (percentage).

Post-operative complications were reported in three patients (27.3%). These included one surgical site infection (9.1%) and two cases of cystitis (18.2%). All three postoperative complications were graded as CD grade 2 and treated with antibiotics. The median hospital stay was 48 (24–96) h. After the final histological results, four patients underwent a second intervention to complete the surgical staging, three by subsequent vNOTES and one by conventional laparoscopy (Table 3).

**Table 3:** Patients with restaging surgery.

No.	Frozen section	Surgical restaging^b^	Definitive histology	TNM	FIGO
5	No	vNOTES omentectomy, peritoneal biopsies	12 mm adult granulosa cell tumor right ovary	pT1a	IA
6	No	vNOTES omentectomy, peritoneal biopsies	3 mm intracystic immature teratoma right ovary	pT1a	IA
8	BOT	Conventional laparoscopy: TLH+contralateral adnexectomy, +pelvic and lomboaortic lymphadenectomy	Invasive mucinous ovarian carcinoma with 2 mm infiltrating component	pT1c1	IC1
9	STIC^a^	vNOTES omentectomy and peritoneal biopsies	Left tubal STIC	pT1a	IA

BOT, borderline ovarian tumors; STIC, serous tubal intraepithelial carcinoma; TLH, total laparoscopic hysterectomy.

^a^ Histology from previous surgery.

^b^ Second intervention to complete surgical staging.

Adjuvant chemotherapy was administered in four patients (36.4%), one of whom received palliative chemotherapy for relapsed pancreatic disease. The median time from surgery to adjuvant therapy was 23 (19–31) days. In this series, no evidence of recurrence was observed, with a median follow-up time of 13.4 (6–39) months. One patient (9.1%) died of metastatic pancreatic cancer one year after the surgery. The final histopathological diagnoses are summarized for each patient in Table 1.

## 4 Discussion

The role of MIS in gynecological oncology has undergone progressive development. This has involved introducing both conventional and robot-assisted laparoscopic techniques, which have demonstrated their feasibility and efficacy in staging and treating uterus-confined endometrial cancer (13, 22, 23). In the case of early-stage ovarian cancer, the latest international guidelines recommend midline laparotomy as the standard procedure. The rationale behind this is that the open surgery allows an accurate abdominal exploration and a reduced risk of rupture of the primary tumor. Nevertheless, the laparoscopic and robotic approaches are often used worldwide for surgical staging of BOTs and early-stage ovarian cancer, and some studies have shown better surgical outcomes and no difference in recurrence rates or survival for those who received minimally invasive vs. open surgical staging (5, 24–27). However, the oncologic outcomes remain a topic of debate, lacking sufficient high-quality evidence to change current guidelines (2, 4, 27, 28). To date, only a few publications report a vNOTES approach in the management of ovarian cancer (14, 17, 18).

According to the current guidelines of the European Society of Gynaecological Oncology (ESGO), surgical management of Stage I–II ovarian cancer must include a total hysterectomy and bilateral salpingo-oophorectomy or fertility-sparing surgery (unilateral salpingo-oophorectomy) in selected patients desiring fertility. Peritoneal washings or cytology, taken before manipulation of the tumor, and peritoneal biopsies with at least infracolic omentectomy are also recommended (1). Since omentectomy via vNOTES has been proven to be feasible (14, 18), in the case of intraoperative diagnosis of BOTs or early-stage ovarian cancer, surgical staging through the same vaginal incision is possible.

Many early-stage ovarian cancer diagnoses are made postoperatively on lesions initially presumed benign (29). The accuracy of frozen section varies between 82% and 88% for BOTs and malignant tumor with most discordancy encountered for younger, premenopausal women, early-stage ovarian malignancies and mucinous histology (30). In our series, five patients have a BOTs identified intraoperatively through frozen section analysis. Two out of five patients were reclassified upon final postoperative histopathology as stage IIB low-grade serous ovarian carcinoma and stage IC1 invasive mucinous ovarian carcinoma. We diagnosed one case of serous tubal intraepithelial carcinoma (STIC) during the surgery for endometrioid endometrial carcinoma. One serous BOT diagnosis was made prior to referral to our institution. The remaining four cases were diagnosed postoperatively. Three had small intraovarian non-epithelial cancers, including one immature teratoma and two adult granulosa cell tumors, while one patient was diagnosed with high-grade serous carcinoma.

A further challenge of the MIS approaches in early-stage ovarian cancers is the ability to perform a complete pelvic and paraaortic lymphadenectomy. For clinical stage I and low-risk invasive ovarian tumors such as mucinous, malignant germ cell, and sex cord-stromal tumors as well as for BOTs and STIC, the systematic lymphadenectomy is not recommended (31). The survival benefit of complete staging with lymphadenectomy in early-stage epithelial ovarian cancer has not been confirmed in prospective trials (29), though it is known that 10%–15% of cases are upstaged due to nodal involvement (32) and require adjuvant treatments. Lymphadenectomy might be subsequently omitted if an occult positive lymph node will not influence the adjuvant treatment allocation. We performed only one subsequent retroperitoneal pelvic and paraaortic lymphadenectomy for a stage IA mucinous invasive carcinoma due to a minimal infiltrative invasive component in a majoritarian expansile tumor.

We hypothesize that both the vNOTES technique for pelvic lymphadenectomy and paraaortic lymphadenectomy can be successfully applied to early-stage ovarian malignancies. The vNOTES approach for pelvic lymphadenectomy was first described in 2014, with further validation by other authors (33–35). Additionally, in 2024, a hybrid technique combining vNOTES with a single-port retroperitoneal approach for pelvic and infrarenal paraaortic lymphadenectomy was reported (36). For patients diagnosed intraoperatively with early-stage invasive ovarian cancer requiring both pelvic and paraaortic lymphadenectomy, a vNOTES hybrid approach, may be an option (36–38). If restaging is required, the absence of an abdominal peritoneal scar after retroperitoneal vNOTES can simplify the successive procedures.

In patients with complex surgical history and suspected severe intra-abdominal adhesions vNOTES approach allows pelvic exploration, hysterectomy and adnexectomy if needed, with less risk of organ damage. In one of our patients, we diagnosed a mucinous BOT and a peritoneal relapse of pancreatic cancer after a previous Wipple procedure. Less than 3 weeks after the surgery the patient started palliative chemotherapy.

One limitation of VNOTES is the restricted accessibility of certain anatomical regions, including the posterior costodiaphragmatic recesses, the Morrison's pouch, the lesser omentum and the mesenteric root. Nevertheless, these regions are challenging to explore also by conventional laparoscopy. Furthermore, the visualization of the vesico-uterine peritoneum during VNOTES adnexal surgery is hindered by the presence of the uterus. A hybrid approach with a transabdominal trocar can effectively address this challenge. Ghezzi et al. emphasized that isolated metastases in these specific areas are extremely rare (39). Despite these limitations, several studies have shown no significant differences in surgical outcomes, recurrence rates or survival between patients undergoing minimally invasive vs. open surgical staging for patients with early-stage ovarian cancer (4–6).

The duration of vNOTES and standard laparoscopic procedures for early stages of adnexal malignancies seems equivalent. Data in the literature is heterogeneous, comparing the time of the open vs. MIS approach with no clear advantage for one or another. The surgeons' experience may be the main factor influencing the operating time (4, 5, 28, 40). The blood loss reported in our series is low and consistent with existing literature (4, 5, 28, 40).

In our series, perioperative complication rates were low. No intraoperative complications were noted, except for one case of minimal ovarian spillage during adnexal extraction in endobag (9.1%). Tumor spillage might occur even in large laparotomies, raising the possibility that aggressive biology associated with more adherent and fragile tumors may be responsible for rupture more than the surgical approach (29). This is crucial as spillage can lower survival rates and is associated with an upstaging of the tumor (40–42). Some studies suggest a higher risk of cyst rupture with laparoscopic cystectomy, which may be reduced if adnexectomy is performed rather than cystectomy (43). Evidence on spillage risk with vNOTES is scarce but appears similar to the laparoscopic approach (8, 44, 45).

Lower rates of all types of complications have been reported with the vNOTES approach in benign indications, ranging from 2.5% to 4.1% (46–48). Laparoscopy has been demonstrated to significantly reduce the duration of hospitalization compared to laparotomy (49), a finding consistent with our series, which had a median hospital stay of 48 (24–96) h. Postoperative complications related to surgery were minimal in our cohort, with one case surgical site infection (9.1%) and two cases of cystitis (18.2%), all successfully treated with antibiotics.

In the vNOTES approach, the single vaginal scar can improve the post-operative recovery. Fast recovery is particularly important for the management of malignant cases, allowing for the earlier administration of adjuvant treatments. In our series, the median time from surgery to adjuvant therapy was 23 (19–31) days. Present recommendations endorse starting adjuvant treatments 28–42 days after the surgery (50).

Abdominal port site metastases has been an important concern in MIS for intra-abdominal malignancies. The vNOTES approach offers an advantage, particularly in patients requiring hysterectomy, by eliminating the need for additional abdominal incisions. For adnexal surgery, vNOTES limits this concern by the presence of a single vaginal incision. Furthermore, vNOTES allows extraction of masses of up to 6–7 cm without the need for morcelation or puncture. For bigger sizes, adnexal mass extraction after puncture in surgical bags is possible with the same approaches as with conventional laparoscopy (8). Baekelandt and al. have recently described a technique for bagging a 20 cm BOTs via vNOTES without spillage (19).

With vNOTES access, vaginal post-site metastasis becomes a significant concern for patients with ovarian cancer. As described by Chitrathara et al., the vaginal vault is a potential site of recurrence in ovarian cancer following laparoscopic treatment and is associated with a worse prognosis compared to other port-site recurrences (51). In their retrospective series, all specimens were extracted vaginally without an endobag for presumed benign lesions. In our series, all patient underwent surgery through a transvaginal access platform protecting both the vaginal wall and the colpotomy site. If morcellation or puncture of ovarian mass were necessary, we used additional layers of protection provided by Inzii retrieval system or Alexis contained extraction system to minimize the risk of contamination.

We acknowledge limitations in this study, notably the small sample size, which limits comparative analysis with other methods. Additionally, case heterogeneity and short follow-up as well as single-center setting with only one oncogynecological surgeon reduce generalizability. However, our focus on early surgical outcomes supports the feasibility of vNOTES for highly selected early-stage ovarian cancer patients, with potential benefits in perioperative morbidity and quality of life.

In conclusion, despite the limited cohort size, our findings indicate the technical feasibility of vNOTES for highly selected patients with early-stage ovarian cancer. Further research with larger cohorts and extended follow-up is needed to assess the long-term oncological outcomes and safety of this technique.

## Data Availability

The original contributions presented in the study are included in the article/Supplementary Material, further inquiries can be directed to the corresponding author.
